# Myocardial Work in Children With Hypertrophic Cardiomyopathy: Longitudinal Evaluation and Prognostic Implications

**DOI:** 10.1016/j.jacadv.2025.101885

**Published:** 2025-06-17

**Authors:** Xander Jacquemyn, Rebbeca Dryer, Kyla Cordrey, Rita Long, David A. Danford, Shelby Kutty, Benjamin T. Barnes

**Affiliations:** a The Blalock-Taussig-Thomas Pediatric and Congenital Heart Center, Department of Pediatrics, Johns Hopkins School of Medicine, Johns Hopkins University, Baltimore, Maryland, USA; b Department of Cardiovascular Sciences, KU Leuven, Leuven, Belgium

**Keywords:** clinical outcomes, echocardiography, global longitudinal strain, hypertrophic cardiomyopathy, pediatric cardiology, myocardial work

## Abstract

**Background:**

Myocardial work (MW) predicts adverse outcomes in adults with hypertrophic cardiomyopathy (HCM), yet pediatric data are lacking.

**Objectives:**

The aim of the study was to describe longitudinal changes in MW and evaluate associations with adverse outcomes in a pediatric population.

**Methods:**

A total of 74 patients with HCM (11.9 years [7.7-14.5], 50% males) were included. MW indices—global work index (GWI), global constructive work (GCW), global wasted work, and global work efficiency (GWE)—were measured and compared with a family history group (FH) (n = 72) (defined as having a first-degree relative with HCM, a second-degree relative with sudden cardiac death (SCD), or a pathogenic mutation without positive phenotype) and healthy controls (n = 50). The primary outcome was a composite endpoint encompassing all-cause mortality, SCD, aborted SCD, appropriate implantable cardioverter-defibrillator discharge, and sustained ventricular tachycardia.

**Results:**

MW indices differed significantly between groups at baseline. In HCM patients, GWI, GCW, and GWE were lower than in FH (pairwise *P* = 0.012, *P* < 0.001, and *P* = 0.001, respectively), while only GCW and GWE were significantly lower in HCM compared to healthy control (both pairwise *P* < 0.001). During follow-up (4.9 years [2.9-8.8]), patients with HCM showed significant decreases in GWI and GCW (*P* = 0.002 and *P* = 0.001), while global wasted work and GWE did not show significant changes (*P* = 0.665 and *P* = 0.126). In contrast, FH patients exhibited stable MW indices over time. Lower GWI and GCW were positively associated with the composite endpoint (both *P* < 0.001).

**Conclusions:**

In pediatric HCM, MW declines over time and is linked to adverse outcomes but remains primarily a research tool, with no superior risk stratification compared to global longitudinal strain.

Hypertrophic cardiomyopathy (HCM) is a common inherited cardiovascular disease, with a prevalence of at least 1 in 500 in the general population,^1^ thereby affecting as many as 20 million people globally.^2^ Despite ventricular hypertrophy, HCM patients typically present with normal or elevated left ventricular (LV) ejection fraction.^3^ However, systolic strain measured using echocardiographic speckle-tracking or cardiac magnetic resonance feature-tracking is often abnormal and correlates with the severity of exercise impairment.^4^ Therefore, systolic LV dysfunction without depressed ejection fraction is common in HCM at rest and likely coexists with load-induced dynamic systolic dysfunction capable of producing symptoms.^4^ Myocardial work (MW) is a means to evaluate LV systolic function, derived from pressure-strain loops based on 2D speckle tracking echocardiography (STE) and noninvasive brachial artery cuff pressure measurement.^5^ MW has been proposed as an accurate and reproducible index of LV systolic function, which accounts for both afterload and myocardial deformation.^5^^,^^6^ This new technique allows for a better understanding of the relationship between LV remodeling and increased wall stress under different loading conditions.^6^^,^^7^ Recently, MW has shown potential utility for quantification of LV systolic function in adult patients with HCM.^8^ It has been demonstrated that global constructive work (GCW) is significantly reduced compared with healthy controls (HCs) and that impaired MW is associated with significant fibrosis on cardiac magnetic resonance imaging.^9^^,^^10^ More recently, it has also been demonstrated to be significantly associated with a worse long-term outcome in this population.^8^ These associations have not been investigated thoroughly in the young, so we aimed to evaluate MW changes over time and identify whether MW was associated with adverse outcomes.

## Methods

### Study population

In this retrospective in-hospital cohort study, we identified patients with HCM from an ongoing clinical registry at our institution between 2008 and 2023. HCM was diagnosed according to the 2020 American Heart Association/American College of Cardiology Guideline for the Diagnosis and Treatment of Patients With Hypertrophic Cardiomyopathy.^11^ A threshold of Z-score >2.5 maximal end-diastolic wall thickness anywhere in the left ventricle was used in asymptomatic children with no family history (FH), whereas for children with a definitive FH or a positive genetic test, a threshold of Z-score >2 was used. Obstructive HCM was defined by a Doppler-derived peak left ventricular outflow tract (LVOT) gradient of ≥30 mm Hg.^11^ From the same clinical registry, patients who had either a positive FH (defined as having a first-degree relative with HCM or a second-degree relative with a history of sudden cardiac death [SCD]) or a pathogenic mutation for HCM but did not meet the clinical diagnosis or phenotype criteria for HCM (defined above) were included as a comparison group (FH). Finally, 50 HCs with a similar age and sex distribution were selected and enrolled from a prospective group of patients. The HC group underwent echocardiograms for clinical indications of heart murmur, atypical chest pain, syncope, or a FH of congenital heart disease. All HCs had normal cardiac structure and function upon echocardiography. Patients without adequate image quality for myocardial deformation analysis or the absence of noninvasive blood pressure (BP) values required for calculation of MW were excluded. Clinical data were collected from retrospective chart review, and the first echocardiogram available for analysis was defined as baseline. The study was approved by the Johns Hopkins Medicine Institutional Review Board with waiver of informed consent due to the retrospective nature of the study.

### Clinical assessment and echocardiography measurements

Height and weight were recorded, and body surface area (BSA) and body mass index were calculated for all patients. BP was measured using an automated BP cuff in the sitting position immediately prior to echocardiographic examination. An appropriately sized cuff was chosen, and BP was measured in the right arm, unless otherwise indicated in standard measurement guidelines. Definitions for hypertension followed the American Academy of Pediatrics guidelines.^12^ Standard imaging windows and measurements were made according to the American Society of Echocardiography guidelines following a standardized protocol using Vivid E9 scanners (GE HealthCare).^13^ LV internal dimensions, interventricular septal thickness, and LV posterior wall thickness were measured in the parasternal short-axis view at both end-diastole and end-systole. Maximal LV wall thickness was evaluated at varying levels from base to apex. LV ejection fraction (LVEF) was calculated using Simpson's biplane measurement in the apical four- and two-chamber views. LV fractional shortening was obtained from M-mode imaging in the parasternal short-axis view. LVOT pressure gradients were calculated through continuous-wave Doppler-obtained peak and mean velocity (V_max_ and V_mean_, respectively) using the simplified Bernoulli equation. Global longitudinal strain (GLS) was measured using STE in the apical 4-chamber, 2-chamber, and 3-chamber views. Analysis of images was performed in EchoPAC software (version 202, GE Healthcare) through automatic tracking of the endocardial contour, utilizing automatic functional imaging provided by the software. Tracking was manually verified, and the region of interest was evaluated to confirm inclusion of the entire LV myocardial thickness.

### Myocardial work measurements

MW analysis was performed using a combination of strain data and noninvasive estimated LV pressure curves based on brachial cuff BP measurements. MW was obtained by entering the measured BP as well as valvular event timing including mitral valve closure, aortic valve opening, aortic valve closure, and mitral valve opening. Additionally, for patients with LVOT obstruction, we performed a corrected MW method, which was previously validated using cardiac catheterization measurements. This method utilizes the aortic valve mean gradient from the continuous-wave Doppler signal and the brachial cuff systolic blood pressure (SBP), and the sum of these values was substituted for the MW analysis to correct for LV systolic pressure in the setting of obstructive HCM.^14^ Noninvasive pressure-strain loops were then calculated. MW indices were defined as follows: global work index (GWI) was defined as total work within the area of the pressure-strain loop, calculated from mitral valve closure to mitral valve opening. GCW was defined as the work performed during shortening in systole in addition to negative work during lengthening in isovolumetric relaxation. Global wasted work (GWW) was defined as the negative work performed during lengthening in systole in addition to work performed during shortening in isovolumetric relaxation. Global work efficiency (GWE) was defined as: GCW/(GCW + GWW).

### Clinical outcomes

The primary outcome was a composite endpoint encompassing all-cause mortality, SCD, aborted SCD, appropriate implantable cardioverter-defibrillator (ICD) discharge, and sustained ventricular tachycardia (VT). Aborted SCD was defined as the successful resuscitation from cardiac arrest with documented ventricular arrhythmias, while appropriate ICD discharge referred to the administration of shock or antitachycardia pacing for ventricular arrhythmias.

### Statistical analysis

Between-group differences were compared using analysis of variance (ANOVA) for normally distributed variables and Kruskal-Wallis for non-normally distributed variables, as appropriate. Post hoc pairwise comparisons were adjusted for multiple testing using the Tukey correction method and Benjamini-Hochberg method, as appropriate.

Univariable and multivariable linear mixed-effects regression was used to understand the association between independent demographic and echocardiographic variables and the dependent MW indices. Using a stepwise elimination approach, the best multivariable model was determined from the Akaike information criterion. Goodness of fit was evaluated by a conditional coefficient of determination (Nakagawa conditional R^2^). To evaluate the effect of time and group differences on MW indices, linear mixed-effects models were used, with time since baseline and group (FH or HCM) as fixed effects. An interaction term (time ∗ group) was included to test whether the rate of change over time differed between groups. To account for repeated measures within individuals, a random intercept was included for each patient. A subgroup analysis was performed to assess whether excluding HCM phenocopies (heterogeneous HCM etiologies) influenced the associations between groups. Additionally, sensitivity analyses were conducted to explore the potential influence of medication usage (calcium-channel blockers, beta-blockers, and septal reduction therapy [SRT] on these associations. Relative change between LVEF, GLS, and MW was calculated using per-patient time profiles obtained from fixed linear-effects regression, and standardized coefficients were compared between patients using the paired t-test or Wilcoxon signed-rank test. Time-to-event data, using the first echocardiography assessment as the baseline, were plotted using the Kaplan-Meier method. Cox regression models using baseline and time-dependent covariates were used to assess the relationship between these variables and the composite endpoint. Patient data were right-censored at the last clinic visit. Multivariable models were performed, adjusting for age, sex, and BSA. A 2-tailed *P* value <0.05 was considered statistically significant. All analyses were performed with R Statistical Software (version 4.4.0) and Python (version 3.11.3).

## Results

### Study population

**Figure 1:**
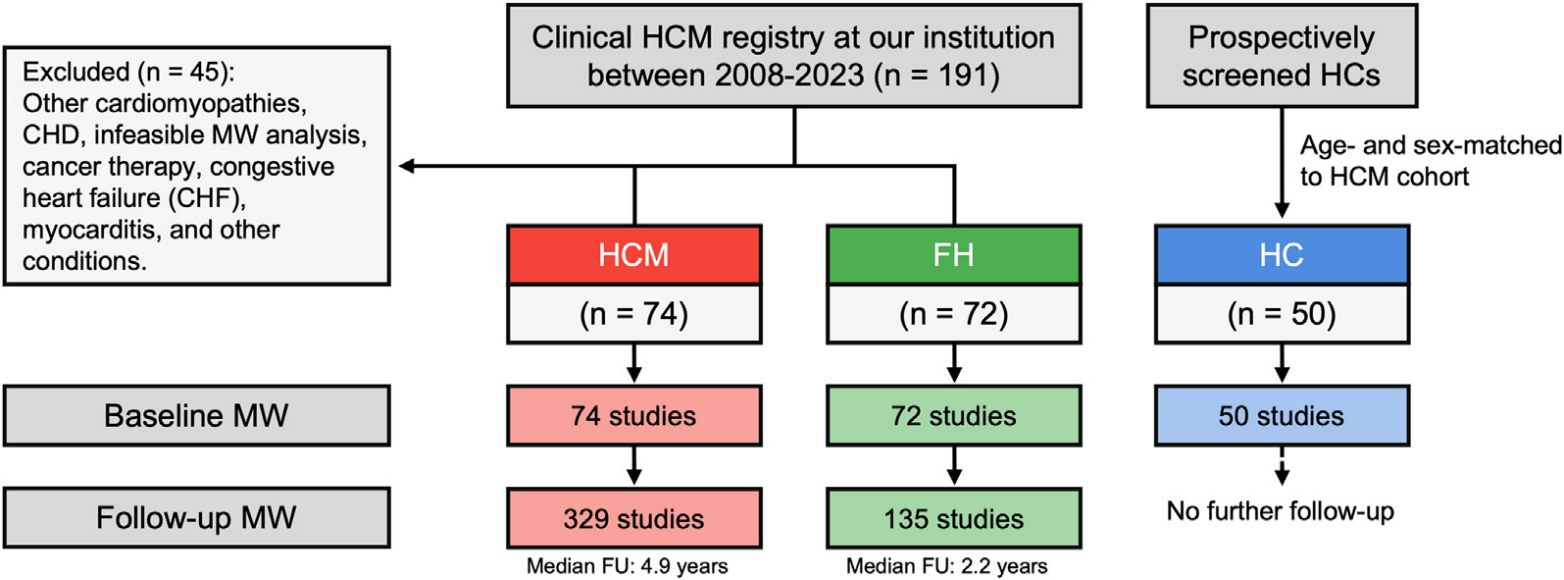
Flowchart of the Study Population CHD = congenital heart disease; FH = family history; FU = follow-up; HC = healthy control; HCM = hypertrophic cardiomyopathy; MW = myocardial work.

The study population encompassed 74 pediatric patients diagnosed with HCM and 72 patients with a positive FH or pathogenic mutation for HCM, but without a clinical diagnosis or phenotype of HCM, who were included as a comparison group (FH) (Figure 1). In the FH group, 85.7% had a first-degree relative with HCM, while 27.8% had either a first- or second-degree relative with a history of SCD. Furthermore, 41 (56.9%) of the FH patients were siblings of the HCM patients included in the study. Lastly, 50 HCs were included. The groups were well matched and had similar sex (54%, 60%, and 50% males in HCs, FH, and HCM respectively, ANOVA *P* = 0.496), age (12.5 [10.0-15.7], 13.2 [9.35-16.1], and 11.9 [7.72-14.5], respectively, ANOVA *P* = 0.165), and BSA (1.46 ± 0.49, 1.47 ± 0.45, and 1.30 ± 0.58, respectively, ANOVA *P* = 0.097). Clinical characteristics of the study population (FH and HCM) are summarized in Table 1. Genetic testing was more commonly performed in the HCM group, with 74.3% undergoing testing compared to 40.3% in the FH group (*P* < 0.001). Results from genetic testing are described in Table 1. In both groups, sarcomeric mutations were most common. In the HCM group, other etiologies included, for instance, 9 patients with Noonan's syndrome, 3 with AMP-kinase mutations, 2 with glycogen storage disorders, and 1 with a carnitine disorder.

**Table 1:** Baseline Characteristics and Echocardiography Parameters

	FH (N = 72)	HCM (N = 74)	*P* Value
Demographics			
Male	43 (59.7)	37 (50.0)	0.311
Age at first echo, y	13.2 [9.35-16.1]	11.9 [7.7-14.5]	0.069
BSA, m^2^	1.47 (0.45)	1.3 (0.6)	0.053
SBP, mm Hg	114 [105-120]	113.0 [105.0-121.0]	0.980
DBP, mm Hg	63.0 [56.8-68.0]	59.0 [55.0-65.0]	0.094
Genetic testing (n = 84)			
Pathogenic mutation	23 (79.3)	44 (77.2)	0.999
Sarcomeric	20 (90.9)	25 (56.8)	0.020
MYH7	6 (20.7)	8 (14.0)	
MYBPC3	6 (20.7)	11 (19.3)	
TNNT2	1 (3.5)	1 (1.8)	
TPM1	1 (3.5)	2 (3.5)	
MYL3	2 (6.9)	1 (1.8)	
Malformation	1 (4.6)	10 (22.7)	0.191
Metabolism	0 (0.0)	6 (13.6)	0.258
Echocardiography			
IVSd, cm	0.81 [0.72-0.94]	1.24 [0.84-1.78]	<0.001
IVSd, Z-score	−0.15 [−0.90 to 0.42]	3.03 [1.46-6.42]	<0.001
LVPWd, cm	0.76 [0.70-0.88]	0.98 [0.74-1.24]	<0.001
LVPWd, Z-score	−0.20 [−0.93 to 0.45]	2.10 [0.37-3.50]	<0.001
Septal/LVPW thickness ratio	1.08 [0.96-1.17]	1.15 [1.01-1.68]	0.001
MLVWT, cm	1.26 [1.16-1.43]	1.67 [1.32-2.11]	<0.001
LVM, g	98.7 [76.9-153]	162 [97.3-241]	0.001
LVMi, g/m^2.7^	35.6 [30.2-42.8]	57.4 [45.6-82.9]	<0.001
LVFS, %	38.8 [35.0-41.4]	41.1 [36.8-51.2]	0.006
LVEF, %	66.8 [60.3-70.4]	68.3 [63.6-72.9]	0.031
LVOT PG, mm Hg	6.07 [5.16-7.24]	8.24 [5.83-13.6]	<0.001
LVOT MG, mm Hg	2.99 [2.49-3.51]	3.78 [2.67-6.13]	0.007
GLS, %	−21.20 [−22.83 to −18.85]	−17.80 [−21.10 to −15.28]	<0.001
GWI, mm Hg%	1,954 [1,768-2,200]	1,786 [1,533-2,081]	0.004
GCW, mm Hg%	2,104 [1,901-2,329]	1,903 [1,574-2,202]	<0.001
GWW, mm Hg%	54.5 [39.8-76.8]	65.0 [46.8-96.5]	0.053
GWE, %	97.0 [96.0-98.0]	95.5 [93.0-97.0]	<0.001

Values are mean ± SD, median [Q25-Q75], or n (%).

BSA = body surface area; d = diastole; DBP = diastolic blood pressure; FH = family history; GCW = global constructive work; GLS = global longitudinal strain; GWE = global work efficiency; GWI = global work index; GWW = global wasted work; HCM = hypertrophic cardiomyopathy; IVS = interventricular septal thickness; LV = left ventricle; LVEF = LV ejection fraction; LVFS = LV fractional shortening; LVID = LV internal dimension; LVM = LV mass; LVMi = LV mass indexed to BSA; LVOT = LV outflow tract; LVPWD = LV posterior wall thickness; MG = mean gradient; MLVWT = maximal LV wall thickness; PG = peak gradient; SBP = systolic blood pressure.

**Figure 2:**
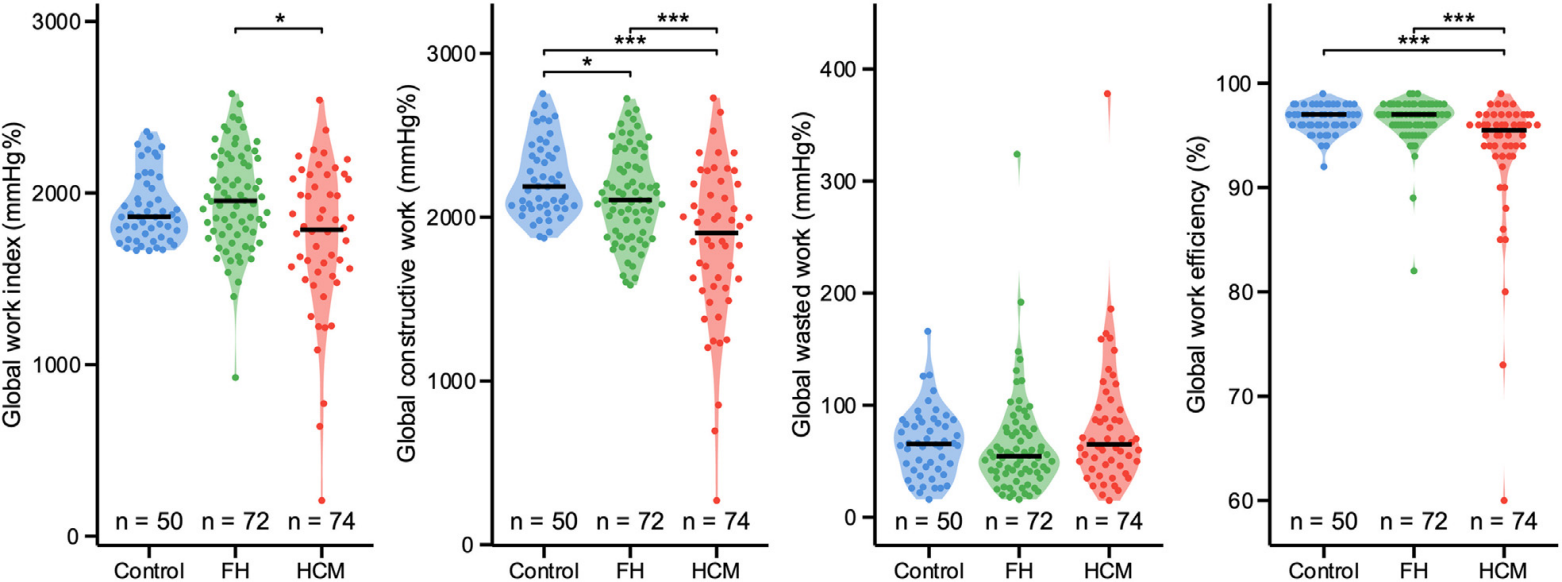
Myocardial Work Indices at Baseline Violin plots displaying the median and distribution of the myocardial work indices. Pairwise comparisons between groups, adjusted for multiple testing using the Tukey correction method or Benjamini-Hochberg method, as appropriate. The symbols signify the following significance level: ∗*P* ≤ 0.05, ∗∗*P* ≤ 0.01, ∗∗∗*P* ≤ 0.001. FH = family history; HCM = hypertrophic cardiomyopathy.

MW indices were significantly different between the groups at baseline (Figure 2).

GWI values were 1,861 [1,747-2,047] mm Hg% in HCs, 1,954 [1,768-2,200] mm Hg% in FH, and 1,786 [1,533-2,081] mm Hg% in HCM (ANOVA *P* = 0.009). Pairwise comparison showed a trend toward lower GWI in HCM vs HC (*P* = 0.064) but no significant difference between FH and HC (*P* = 0.235). Similarly, GCW was lower in HCM (1,903 [1,574-2,202] mm Hg%) compared to FH (2,104 [1,901-2,329] mm Hg%) and HC (2,187 [2,057-2,404] mm Hg%) (ANOVA *P* < 0.001), with a slight increase in HC compared to FH (pairwise *P* = 0.049). GWE was also lower in HCM (95.5% [93.0%-97.0%]) compared to FH (97.0% [96.0%-98.0%]) and HC (97.0% [96.0%-98.0%]) (ANOVA *P* < 0.001); however, pairwise comparisons showed no significant difference between FH and HC (*P* = 0.724). In contrast, GWW did not differ significantly among groups (ANOVA *P* = 0.117). Other echocardiographic characteristics of the study population (FH and HCM) are summarized in Table 1. Eleven HCM patients (14.9%) had LVOT obstruction at baseline (LVOT peak gradient: 64.6 ± 18.9 mm Hg and mean gradient: 24.4 ± 12.5 mm Hg), and in 4 others (5.4%), SRT using myectomy was performed prior to study enrollment, eliminating the LVOT obstruction.

During a median follow-up period of 4.9 years [2.9-8.8], a total of 403 echocardiography assessments were performed in HCM patients (median 5 [2-8] per patient), and 207 echocardiography assessments in FH+ patients (median 3 [1-4] per patient) during a median follow-up period of 2.2 years [0-5.3]. During echocardiographic follow-up, in addition to the 11 patients who had obstructive HCM at baseline, 3 more developed an obstructive HCM phenotype. Additionally, 4 more patients underwent SRT using myectomy. There were 11 patients with an ICD at baseline, and 3 more received an ICD during follow-up.

### Relationship of MW indices to baseline and echocardiographic parameters

Results from univariable and multivariable regression analysis for the MW indices in the HCM group are available in Supplemental Table 1. Multivariable analysis for MW indices showed that GWI increased with SBP and decreased with GLS. Additionally, multivariable analysis demonstrated the best fit using a combination of both absolute IVSd and LVPWd values and Z-scores. GCW showed a similar pattern, with increasing values with SBP and decreasing values with GLS.

### Longitudinal changes in systolic function

**Figure 3:**
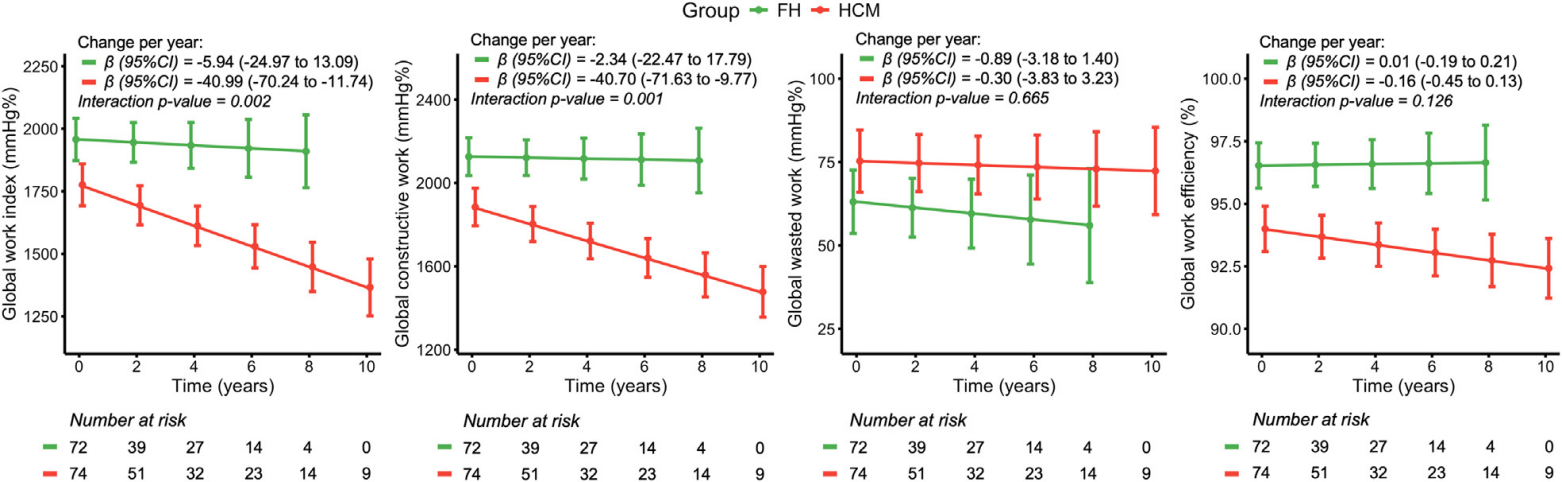
Mean Change of Myocardial Work Indices Over Time MW indices are plotted with the 95% CI at the respective time intervals. Patients with HCM (red) are compared to patients with a positive family history or a pathogenic mutation without the HCM phenotype (green). The yearly change in MW indices is expressed as β (95% CI). FH = family history; GCW = global constructive work; GWE = global work efficiency; GWI = global work index; GWW = global wasted work; HCM = hypertrophic cardiomyopathy; MW = myocardial work.

The trajectories of MW indices are shown in Figure 3. Patients without an HCM diagnosis (FH) demonstrated stable MW indices during follow-up. The yearly change in MW indices, expressed as β (95% CI), along with the interaction *P* values are presented in Figure 2.

In a subgroup analysis, we excluded confirmed phenocopies of HCM, including lysosomal storage disorders (n = 1), glycogen storage disorders (n = 1), PRKAG2 cardiomyopathy (n = 3), RASopathies (mainly Noonan syndrome, n = 9; cardiofaciocutaneous syndrome, n = 1), mitochondrial diseases (n = 2), and other rare conditions (n = 4). The associations of the yearly change in MW indices between the groups remained unchanged (Supplemental Figure 1).

In further sensitivity analyses of HCM patients, adjusted for potential confounders, medication usage significantly influenced MW indices (Supplemental Table 2). Calcium-channel blockers were associated with lower baseline GWI and GWC but slower rates of deterioration. No significant effects were found for beta blockers. Similarly, SRT was linked to lower baseline GWI and slower deterioration in GWI and GCW. The individual components of MW (GLS, SBP, and diastolic BP) were assessed over time, with only GLS and diastolic BP showing significant interaction effects during the follow-up period (Supplemental Figure 2).

To evaluate whether MW could provide incremental value over GLS by demonstrating earlier subclinical dysfunction, we compared the standardized slopes of individual patients with HCM. In comparison to LVEF, only GLS (*P* < 0.001), GWI (*P* = 0.003), and GCW (*P* = 0.011) provided incremental value. When comparing GLS to both GWI and GCW, no significant differences were observed (standardized β coefficient: −0.028 vs −0.034, *P* = 0.486 and −0.028 vs −0.032, *P* = 0.228, respectively).

### Clinical outcomes

**Figure 4:**
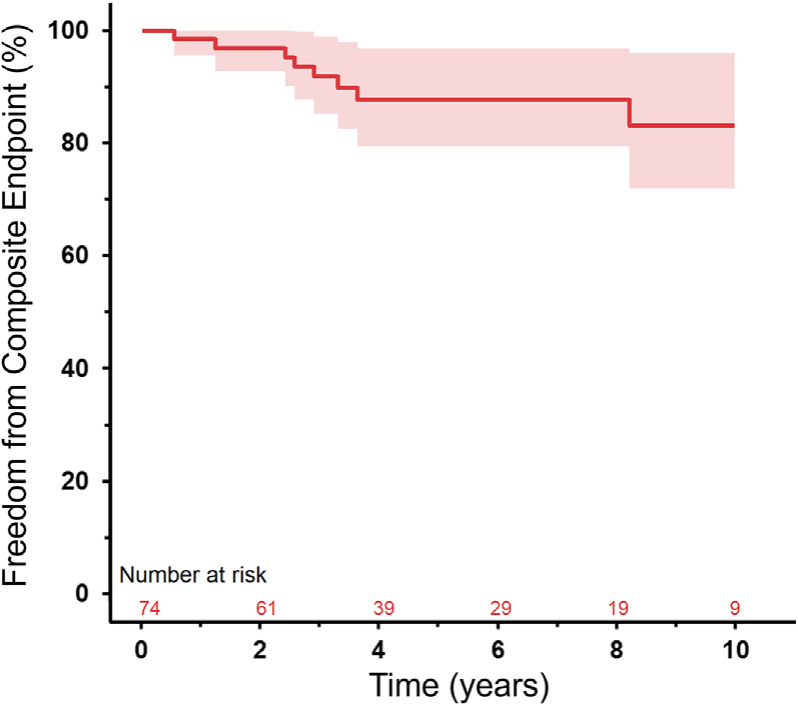
Kaplan-Meier Survival Curves Depicting Time to Composite Endpoint-Free Survival in Patients With Hypertrophic Cardiomyopathy Kaplan-Meier survival curves and 95% CI depicting time to composite endpoint-free survival (all-cause mortality, sudden cardiac death, aborted sudden cardiac death, appropriate ICD discharge, and sustained VT). ICD = implantable cardioverter-defibrillator; VT = ventricular tachycardia.

During the median follow-up period of 4.9 years [2.9-8.8], 11 patients (15.5%) with HCM reached the composite endpoint (for a combined total of 15 events) (Supplemental Table 3, Figure 4). Five patients received an appropriate ICD discharge, 3 patients had sustained VT, 4 had aborted SCD, and 3 patients died. The cause of death was cardiac in all 3 cases. Two patients had multiple events; both patients first had an aborted SCD event, followed by appropriate ICD discharge for sustained VT later during follow-up. None of the baseline variables were associated with the composite endpoint. On echocardiographic evaluation, at any given moment, only GLS (HR: 1.13 [1.03-1.24] per 1% deterioration, *P* = 0.008), GWI (HR: 0.98 [0.97-0.99] per 10 mm Hg% increase, *P* < 0.001), and GCW (HR: 0.98 [0.97-0.99] per 10 mm Hg% increase, *P* < 0.001) were associated with the composite endpoint (Table 2). The proportional hazards assumption was satisfied for all variables (Supplemental Table 4). Models adjusted for age, sex, and BSA are provided in Table 3. No events occurred in the FH group.

**Table 2:** Cox Regression Model and Time-Dependent Cox Regression Model for the Risk of Outcomes in Patients With HCM

Baseline Variables	Outcome (n = 11)		
	HR (95% CI)	*P* Value	C-Statistic
Male	1.49 (0.40-5.54)	0.553	0.570
Age at first echo, y	1.04 (0.91-1.18)	0.572	0.572
BSA, m^2^	1.25 (0.40-3.96)	0.703	0.556
History of syncope	2.70 (0.52-14.00)	0.276	0.640
Hypertension	0.39 (0.08-1.87)	0.204	0.556
Obstructive HCM	2.70 (0.52-14.00)	0.276	0.581
Pathogenic mutation	1.52 (0.32-7.20)	0.586	0.568

Time-Dependent Variables	Outcome (n = 15)		
	HR (95% CI)	*P* Value	C-Statistic
SBP, mm Hg	0.98 (0.94-1.02)	0.280	0.613
DBP, mm Hg	0.94 (0.88-1.01)	0.088	0.651
LVOT PG, mm Hg	1.01 (0.97-1.04)	0.689	0.569
LVOT MG, mm Hg	1.02 (0.95-1.10)	0.559	0.500
LVIDd, Z-score	0.83 (0.87-1.67)	0.262	0.608
IVSd, Z-score	1.05 (0.97-1.14)	0.210	0.688
LVPWd, Z-score	1.03 (0.81-1.17)	0.787	0.566
MLVWT, cm	1.58 (0.76-3.30)	0.221	0.659
LVMi, g/m^2.7^	1.00 (1.00-1.01)	0.218	0.638
LVFS, %	0.96 (0.91-1.01)	0.095	0.666
LVEF, %	0.99 (0.94-1.04)	0.670	0.689
GLS, %	1.13 (1.03-1.24)	0.008	0.797
GWI, per 10 mm Hg%	0.98 (0.97-0.99)	<0.001	0.833
GCW, per 10 mm Hg%	0.98 (0.97-0.99)	<0.001	0.833
GWW, per 10 mm Hg%	0.90 (0.74-1.09)	0.274	0.658
GWE, %	0.99 (0.91-1.08)	0.842	0.478

BSA = body surface area; d = diastole; DBP = diastolic blood pressure; GCW = global constructive work; GLS = global longitudinal strain; GWE = global work efficiency; GWI = global work index; GWW = global wasted work; HCM = hypertrophic cardiomyopathy; IVS = interventricular septal thickness; LV = left ventricle; LVEF = LV ejection fraction; LVFS = LV fractional shortening; LVID = LV internal dimension; LVMi = LV mass indexed to BSA; LVOT = LV outflow tract; LVPWd = LV posterior wall thickness; MG = mean gradient; MLVWT = maximal LV wall thickness; PG = peak gradient; SBP = systolic blood pressure.

**Table 3:** Adjusted Cox Regression Models for the Risk of Outcomes in Patients With HCM for the Major Echocardiographic Variables

Time-Dependent Variables	Outcome (n = 15)		
	HR (95% CI)∗	*P* Value	C-Statistic
LVEF, %	1.00 (0.95-1.05)	0.670	0.871
GLS, %	1.14 (1.00-1.31)	0.055	0.833
GWI, per 10 mm Hg%	1.00 (1.00-1.00)	0.010	0.866
GCW, per 10 mm Hg%	1.00 (1.00-1.00)	0.009	0.857
GWW, per 10 mm Hg%	1.00 (1.00-1.00)	0.157	0.678
GWE, %	1.02 (0.92-1.13)	0.717	0.602

BSA = body surface area; GCW = global constructive work; GLS = global longitudinal strain; GWE = global work efficiency; GWI = global work index; GWW = global wasted work; HCM = hypertrophic cardiomyopathy; LVEF = left ventricular ejection fraction.

∗ Models were adjusted for age, sex, and BSA.

## Discussion

The present study demonstrates that MW is significantly impaired in a pediatric HCM population and provides insights into long-term deterioration in this population (Central Illustration). Our findings of impaired MW in HCM, compared to both internal HCs and patients with a positive FH, are consistent when compared to two published HC cohorts.^15^^,^^16^ When compared to these external studies, in our HCM population, more patients had GWI values below the 5th percentile for age at baseline (11.9% to 15.9%) and follow-up (25.9% to 30.4%). Conversely, more patients had GWW values above the 95th percentile at baseline (22.0% and 16.9%), which decreased over time (9.4% and 9.7%). However, in our internal control cohort, no differences in GWW from HCs were observed at baseline. However, these findings may represent conservative estimates due to the smaller sample size in these studies. When compared to a larger international cohort of over 1,000 HCs included in a systematic review,^17^ a greater proportion of our HCM cohort fell below the age-specific reference range: 37.8% for GWI, 51.4% for GCW, and 66.2% for GWE. In contrast, FH patients showed substantially lower proportions, with only 4.2% for GWI, 9.7% for GCW, and 27.8% for GWE falling below the reference ranges. Our results seem plausible, as reductions in noninvasive MW indices have been observed in adults^8^^,^^18^ and pediatric patients^19^ with HCM. Among adults, these findings have been linked to reductions in LV systolic function, the presence of fibrosis,^9^ and other adverse outcomes. For example, GWI, GCW, and GWE are significantly reduced, with increased GWW, especially in areas of maximal wall thickness (reflecting individual differences according to HCM phenotype).^8^

**Central Illustration:**
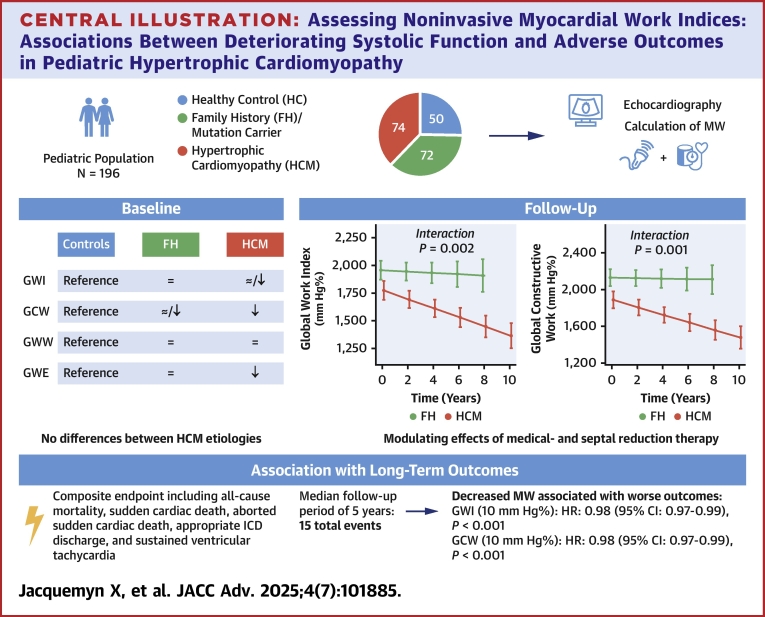
Assessing Noninvasive Myocardial Work Indices: Associations Between Deteriorating Systolic Function and Adverse Outcomes in Pediatric Hypertrophic Cardiomyopathy GCW = global constructive work; GWE = global work efficiency; GWI = global work index; GWW = global wasted work; ICD = implantable cardioverter-defibrillator; MW = myocardial work.

During follow-up of our patients, 11 (15.5%) reached the composite endpoint, and we found that GLS, GWI, and GCW were significantly associated with these adverse events. Yet, based on our findings, the benefits of incorporating MW assessment in clinical practice for risk stratification in pediatric HCM remain largely theoretical and are currently overshadowed by the time-consuming nature of the data acquisition process. Until advancements in technology are streamlined for clinical use, we believe MW remains primarily a focus for research in pediatric HCM rather than a tool ready to be adopted as routine direct patient care. Furthermore, in our cohort, we could not demonstrate that MW indices provide more accurate risk stratification than GLS.

Additionally, long-term follow-up revealed significant annual declines in GWI and GCW in HCM patients, reflecting progressive deterioration of myocardial function when compared to patients without HCM. These findings are particularly significant considering that MW is expected to increase with age.^17^ The results from our study may, therefore, indicate more impaired cardiac function than previously recognized. In select HCM patients, the decline in MW indices was modulated by both pharmacological interventions and SRT, with calcium channel blockers and SRT showing a protective effect by slowing the annual deterioration in GWI and GCW.^20^^,^^21^ However, because of selection bias, a causal relationship between medical therapy or SRT and the observed reduction in deterioration cannot be definitively established. Furthermore, it is reasonable to assume that these patients had already reached a low MW threshold by the time of introduction of therapy, making further declines in systolic function less likely.^20^^,^^21^

### Study limitations

Our study has several limitations, including its retrospective design and a relatively small sample size. Nevertheless, it is one of the first studies on pediatric MW incorporating echocardiographically measured pressure gradients across the aortic valve. Another limitation is that the LV pressure estimation algorithms used in the MW analysis were originally developed and validated in adults^5^ and have not been validated in pediatric populations. Beyond this, a further important consideration in MW assessment is that the “work” the LV has to perform is not solely determined by LV pressure—it is also influenced by geometric changes in LV chamber dimensions and wall thickness.^22^^,^^23^ This is indicated by Laplace’s law [$LV\;wall\;stress\;\left(dynes\;{cm}^{2}\right)=\frac{LV\;pressure\;\cdot \;radius}{2\;\cdot \;LV\;wall\;thickness}$], where LV wall stress is directly proportional to LV pressure and geometric changes in LV chamber dimensions but inversely related to LV wall thickness. Additionally, wall stress is further affected by regional wall curvature, as decreasing curvature (a flatter wall and a larger curvature radius) implies higher regional wall stress. As such, in 2 ventricles with the same stroke volume and BP but different chamber volumes, the larger ventricle will experience higher LV wall stress due to its larger radius. Nevertheless, the pressure-volume loop area (stroke volume multiplied by mean arterial pressure) and pressure-strain loop area (GLS multiplied by mean arterial pressure) are similar and do not reflect the increased “work” that has to be performed by the myocardium in the larger ventricle. One of the proposed solutions is the assessment of work using noninvasive regional LV wall stress instead of LV pressure.^24^^,^^25^ In conclusion, in pediatric HCM, noninvasive measures of MW are commonly depressed at baseline and have a strong tendency to decrease during longitudinal follow-up. Although not shown to have incremental value over GLS, MW indices are strong prognostic markers for adverse events in this population.

Perspectives **COMPETENCY IN MEDICAL KNOWLEDGE:** MW indices are potential markers of early myocardial dysfunction in pediatric HCM, emphasizing their potential role in echocardiographic evaluation.

**TRANSLATIONAL OUTLOOK:** Further research is needed to determine the superiority of MW over GLS in predicting disease progression and adverse outcomes. Future studies should validate pediatric MW by correlating it with invasive hemodynamic measurements.

## Funding support and author disclosures

Mr Jacquemyn was supported by a grant from the 10.13039/100001491Belgian American Educational Foundation. All other authors have reported that they have no relationships relevant to the contents of this paper to disclose.
