# Supplementary material for: Myocardial Work in Children With Hypertrophic Cardiomyopathy: Longitudinal Evaluation and Prognostic Implications
Source: JACC Adv. 2025 Jun 17;4(7):101885. doi: 10.1016/j.jacadv.2025.101885 (PMC12212273; doi:10.1016/j.jacadv.2025.101885)
Supplement: Supplemental Material [file mmc1.pdf]

Supplemental Material

STROBE Statement—Checklist of items that should be included in reports of ***cohort studies***

	Item No	Recommendation	Page
**Title and abstract**	1	(*a*) Indicate the study's design with a commonly used term in the title or the abstract	1
		(*b*) Provide in the abstract an informative and balanced summary of what was done and what was found	2
**Introduction**			
Background/rationale	2	Explain the scientific background and rationale for the investigation being reported	4
Objectives	3	State specific objectives, including any prespecified hypotheses	4
**Methods**			
Study design	4	Present key elements of study design early in the paper	5
Setting	5	Describe the setting, locations, and relevant dates, including periods of recruitment, exposure, follow-up, and data collection	5
Participants	6	(*a*) Give the eligibility criteria, and the sources and methods of selection of participants. Describe methods of follow-up	5
		(*b*) For matched studies, give matching criteria and number of exposed and unexposed	NA
Variables	7	Clearly define all outcomes, exposures, predictors, potential confounders, and effect modifiers. Give diagnostic criteria, if applicable	7
Data sources/ measurement	8*	For each variable of interest, give sources of data and details of methods of assessment (measurement). Describe comparability of assessment methods if there is more than one group	*4*
Bias	9	Describe any efforts to address potential sources of bias	5
Study size	10	Explain how the study size was arrived at	5
Quantitative variables	11	Explain how quantitative variables were handled in the analyses. If applicable, describe which groupings were chosen and why	5
Statistical methods	12	(*a*) Describe all statistical methods, including those used to control for confounding	8
		(*b*) Describe any methods used to examine subgroups and interactions	8
		(*c*) Explain how missing data were addressed	NA
		(*d*) If applicable, explain how loss to follow-up was addressed	8
		(*e*) Describe any sensitivity analyses	NA
**Results**			
Participants	13*	(a) Report numbers of individuals at each stage of study—eg numbers potentially eligible, examined for eligibility, confirmed eligible, included in the study, completing follow-up, and analysed	9-12
		(b) Give reasons for non-participation at each stage	NA
		(c) Consider use of a flow diagram	Fig 1
Descriptive data	14*	(a) Give characteristics of study participants (eg demographic, clinical, social) and information on exposures and potential confounders	Table 1

		(b) Indicate number of participants with missing data for each variable of interest	NA
		(c) Summarise follow-up time (eg, average and total amount)	12
Outcome data	15*	Report numbers of outcome events or summary measures over time	12
Main results	16	(*a*) Give unadjusted estimates and, if applicable, confounder-adjusted estimates and their precision (eg, 95% confidence interval). Make clear which confounders were adjusted for and why they were included	9-12
		(*b*) Report category boundaries when continuous variables were categorized	NA
		(*c*) If relevant, consider translating estimates of relative risk into absolute risk for a meaningful time period	NA
Other analyses	17	Report other analyses done—eg analyses of subgroups and interactions, and sensitivity analyses	NA
**Discussion**			
Key results	18	Summarise key results with reference to study objectives	12-15
Limitations	19	Discuss limitations of the study, taking into account sources of potential bias or imprecision. Discuss both direction and magnitude of any potential bias	12-15
Interpretation	20	Give a cautious overall interpretation of results considering objectives, limitations, multiplicity of analyses, results from similar studies, and other relevant evidence	12-15
Generalisability	21	Discuss the generalisability (external validity) of the study results	12-15
**Other information**			
Funding	22	Give the source of funding and the role of the funders for the present study and, if applicable, for the original study on which the present article is based	Title page

*Give information separately for exposed and unexposed groups.

Note: An Explanation and Elaboration article discusses each checklist item and gives methodological background and published examples of transparent reporting. The STROBE checklist is best used in conjunction with this article (freely available on the Web sites of PLoS Medicine at http://www.plosmedicine.org/, Annals of Internal Medicine at http://www.annals.org/, and Epidemiology at http://www.epidem.com/). Information on the STROBE Initiative is available at http://www.strobe-statement.org.

Table S1. Univariable and multivariable linear mixed-effect models for each of the myocardial work indices across all studies in patients with HCM.

Parameter	GWI (mmHg%)		GCW (mmHg%)		GWW (mmHg%)		GWE (%)	
	Univariable	Multivariable (R^2=0.921)	Univariable	Multivariable (R^2=0.923)	Univariable	Multivariable (R^2=0.586)	Univariable	Multivariable (R^2= 0.754)
Age at echo, years	-36 (-46 – -26), **p <0.001**		-37 (-48 – -26), **p <0.001**		-0.51 (-1.7 – 0.64), p = 0.382		-0.16 (-0.27 – -0.04), **p = 0.008**	
Male	1.8 (-199 – 203), p = 0.986		5.1 (-214 – 225), p = 0.964		-15 (-36 – 6.2), p = 0.172		0.1 (-2.4 – 2.6), p = 0.938	
BSA, m^2	-305 (-439 – -171), **p <0.001**		-350 (-492 – -208), **p <0.001**		-10 (-25 – 4.7), p = 0.185	-26 (-40.7 – -11.2), **p <0.001**	-1.7 (-3.2 – -0.21), **p = 0.027**	
SBP, mmHg	12 (8.2 – 15), **p <0.001**	12.6 (10.9 – 14.2), **p <0.001**	13 (9.9 – 17), **p <0.001**	14.6 (12.8 – 16.3), **p <0.001**	0.63 (0.27 – 1.00), **p = 0.001**	0.54 (0.13 – 0.94), **p = 0.010**	0 (-0.03 – 0.04), p = 0.788	
DBP, mmHg	12 (6.4 – 17), **p <0.001**		15 (9.2 – 20), **p <0.001**		0.75 (0.2 – 1.3), **p = 0.008**	0.68 (0.07 – 1.28), **p = 0.029**	-0.01 (-0.06 – 0.05), p = 0.830	
IVSd, cm	-272 (-354 – -191), **p <0.001**	-284 (-472 – -97), **p = 0.003**	-299 (-384 – -213), **p <0.001**	-298 (-496 – -100), **p = 0.003**	0.11 (-9.3 – 9.5), p = 0.982		-1.4 (-2.3 – -0.47), **p = 0.004**	3.0 (1.0 – 5.1), **p = 0.004**
IVSd, Z-score	-26 (-38 – -14), **p <0.001**	33 (7.6 – 58), **p = 0.035**	-29 (-41 – -16), **p <0.001**	36 (9.8 – 63.1), **p = 0.008**	0.39 (-0.91 – 1.7), p = 0.554		-0.17 (-0.3 – -0.04), **p = 0.013**	-0.43 (-0.69 – -0.16), **p = 0.002**
LVPWd, cm	-368 (-531 - -205), **p <0.001**	372 (95 – 648), **p = 0.009**	-423 (-592 – -253), **p <0.001**	359 (68 – 651), **p = 0.016**	-2 (-20 – 16), p = 0.826		-2.6 (-4.3 – -0.82), **p = 0.004**	
LVPWd, Z-score	-27 (-48 – -5.9), **p = 0.013**	-46 (-78 – -14), **p = 0.005**	-33 (-55 – -11), **p = 0.003**	-51 (-85 – -17), **p = 0.004**	-0.01 (-2.2 – 2.2), p = 0.990		-0.21 (-0.43 – 0.01), p = 0.065	
MLVWT, cm	-220 (-299 – -140), **p <0.001**		-243 (-326 – -160), **p <0.001**		-0.34 (-9.1 – 8.5), p = 0.940		-1.2 (-2 – -0.28), **p = 0.010**	
LVM, g	-1.7 (-2.1 – -1.2), **p <0.001**		-1.8 (-2.3 – -1.4), **p <0.001**		-0.02 (-0.07 – 0.03), p = 0.479		-0.01 (-0.01 – 0.00), **p = 0.002**	
LVMi, $g/m^{2.7}$	-1.4 (-2.4 – -0.5), **p = 0.003**		-1.4 (-2.4 – -0.46), **p = 0.004**		0.03 (-0.07 - 0.13), p = 0.520		-0.01 (-0.02 – 0.00), p = 0.112	
LVOT PG, mmHg	3.6 (0.82 – 6.5), **p = 0.012**	3.4 (2.1 – 4.6), **p <0.001**	2.8 (-0.2 – 5.8), p = 0.069	2.4 (1.1 – 3.8), **p <0.001**	0.47 (0.18 – 0.76), **p = 0.001**	1.66 (0.76 – 2.56), **p <0.001**	-0.02 (-0.05 – 0.01), p = 0.265	
LVOT MG, mmHg	7.1 (0.34 – 14), **p = 0.040**		4.8 (-2.3 – 12), p = 0.188		0.67 (-0.03 – 1.4), p = 0.060	-3.1 (-5.3 – -0.9), **p = 006**	-0.03 (-0.1 – 0.04), p = 0.343	
LVFS, %	6.1 (1.1 – 11), **p = 0.017**		4.7 (-0.53 – 10), p = 0.079		0.42 (-0.1 – 0.94), p = 0.114	0.53 (0.02 – 1.03), **p = 0.043**	0.02 (-0.03 – 0.07), p = 0.514	
LVEF, %	13 (7 – 19), **p <0.001**		13 (6.4 – 19), **p <0.001**		-0.24 (-0.81 – 0.34), p = 0.418		0.13 (0.07 – 0.19), **p = <0.001**	0.07 (0.02 – 0.12), **p = 0.009**
GLS, %	-90 (-97 – -83), **p <0.001**	-90 (-97 – -84), **p <0.001**	-94 (-102 – -86), **p <0.001**	-92 (-99 – -85.3), **p <0.001**	1.2 (-0.04 – 2.5), p = 0.058	2.6 (1.3 – 4.0), **p <0.001**	-0.53 (-0.65 – -0.42), **p = <0.001**	-0.58 (-0.71 – -0.44), **p = <0.001**

Slope coefficients (95% confidence interval) and p-values are presented for each respective model. Conditional R^2 is presented for the multivariable models. Abbreviations: BSA, body surface area; d, diastole; DBP, diastolic blood pressure; GCW, global constructive work; GLS, global longitudinal strain; GWE, global work efficiency; GWI, global work index; GWW, global wasted work; IVS, interventricular septal thickness; LV, left ventricle; LVEF, LV ejection fraction; LVFS, LV fractional shortening; LVM, LV mass; LVMi, LV mass indexed to BSA; LVOT, LV outflow tract; LVPWD, LV posterior wall thickness; MG, mean gradient; MLVWT, maximal LV wall thickness; PG, peak gradient; SBP, systolic blood pressure.

Table S2. Modulating effects in subgroup analysis of patients with HCM.

Modulator	MW	Fixed effects coefficients	β (SE)	P-value
Calcium Channel Blockers	GWI	Intercept	1814.0 (52.8)	<0.001
		CCB	-289.2 (99.2)	0.004
		Time	-45.5 (5.6)	<0.001
		Interaction (CCB*Time)	47.5 (19.2)	0.014
	GCW	Intercept	1924.2 (57.4)	<0.001
		CCB	-301.9 (105.3)	0.004
		Time	-45.3 (7.0)	<0.001
		Interaction (CCB*Time)	49.3 (20.2)	0.015
	GWW	Intercept	75.9 (5.9)	<0.001
		CCB	-0.7 (11.0)	0.951
		Time	-0.2 (0.7)	0.763
		Interaction (CCB*Time)	0.9 (2.1)	0.642
	GWE	Intercept	94.1 (0.7)	<0.001
		CCB	-1.0 (1.1)	0.358
		Time	-0.2 (0.1)	0.015
		Interaction (CCB*Time)	0.1 (0.2)	0.667
Beta blockers	GWI	Intercept	1803.2 (59.7)	<0.001
		BB	-89.7 (75.8)	0.238
		Time	-31.2 (9.9)	0.002
		Interaction (BB*Time)	-11.9 (12.9)	0.355
	GCW	Intercept	1919.1 (64.3)	<0.001
		BB	-114.2 (80.4)	0.156
		Time	-28.4 (10.4)	0.007
		Interaction (BB*Time)	-14.9 (13.6)	0.273
	GWW	Intercept	71.8 (6.6)	<0.001
		BB	10.2 (8.4)	0.225
		Time	0.2 (1.1)	0.854
		Interaction (BB*Time)	-0.8 (1.4)	0.572
	GWE	Intercept	94.2 (0.7)	<0.001
		BB	-0.8 (0.8)	0.323
		Time	-0.2 (0.1)	0.129
		Interaction (BB*Time)	0 (0.1)	0.905
SRT	GWI	Intercept	1803.1 (53.9)	<0.001
		SRT	-254.2 (132.5)	<0.001
		Time	-46.3 (6.5)	0.056
		Interaction (SRT*Time)	61.7 (22.1)	0.006
	GCW	Intercept	1906.5 (58.6)	<0.001
		SRT	-201.2 (141.6)	0.157
		Time	-46.8 (6.8)	<0.001
		Interaction (SRT*Time)	68.6 (23.3)	0.003
	GWW	Intercept	74.3 (5.9)	<0.001
		SRT	14.7 (14.6)	0.316
		Time	-0.1 (0.7)	0.909
		Interaction (SRT*Time)	-0.6 (2.5)	0.809
	GWE	Intercept	94 (0.7)	<0.001
		SRT	-0.7 (1.5)	0.647
		Time	-0.2 (0.1)	0.007
		Interaction (SRT*Time)	0.2 (0.2)	0.350

Coefficients from linear mixed effect models (MW ~ Time*Modulator+ (1 | Patient).
Abbreviations: HCM, hypertrophic cardiomyopathy; BB, beta blocker; CCB, calcium channel blocker; SRT, septal reduction therapy; GWI, Global Work Index; GCW, Global Constructive Work; GWW, Global Wasted Work; GWE, Global Work Efficiency.

Table S3. Outcomes in patients with HCM.

Characteristic	HCM (N=74)
Outcomes	
ICD placement	11 (15.5)
ICD discharge	5 (6.8)
Sustained VT	3 (4.1)
NSVT	8 (10.8)
Aborted SCD	4 (5.4)
SCD	0 (0.0)
Cardiac death	3 (4.1)

Abbreviations: HCM, hypertrophic cardiomyopathy; ICD, implantable cardioverter-defibrillator; NSVT, non-sustained ventricular tachycardia; SCD, sudden cardiac death; VT, ventricular tachycardia.

Table S4. Assessment of Proportional Hazards Assumption in Cox and Time-Dependent Cox Regression Models for Risk of Outcomes in Patients with HCM

**Baseline variables**	***Cox PH, p*-value**
Male	0.434
Age at first echo, years	0.993
BSA, m^2	0.550
History of syncope	0.849
Hypertension	0.374
Obstructive HCM	0.752
Pathogenic mutation	0.155
**Time-dependent variables**	***Cox PH, p*-value**
SBP, mmHg	0.829
DBP, mmHg	0.991
LVOT PG, mmHg	0.937
LVOT MG, mmHg	0.889
LVIDd, Z-score	0.906
IVSd, Z-score	0.104
LVPWd, Z-score	0.372
MLVWT, cm	0.108
LVMi, $g/m^{2.7}$	0.993
LVFS, %	0.827
LVEF, %	0.090
GLS, %	0.151
GWI, per 10 mmHg%	0.371
GCW, per 10 mmHg%	0.492
GWW, per 10 mmHg%	0.378
GWE, %	0.936

Abbreviations: BSA, body surface area – d, diastole – DBP, diastolic blood pressure – GCW, global constructive work – GLS, global longitudinal strain – GWE, global work efficiency – GWI, global work index – GWW, global wasted work – HCM, hypertrophic cardiomyopathy – IVS, interventricular septal thickness – LV, left ventricle – LVEF, LV ejection fraction – LVFS, LV fractional shortening – LVID, LV internal dimension – LVMi, LV mass indexed to BSA – LVOT, LV outflow tract – LVPWD, LV posterior wall thickness – MG, mean gradient – MLVWT, maximal LV wall thickness – PG, peak gradient – SBP, systolic blood pressure

Supplemental Figure 1. Mean change of myocardial work indices over time in non-phenocopies of HCM.

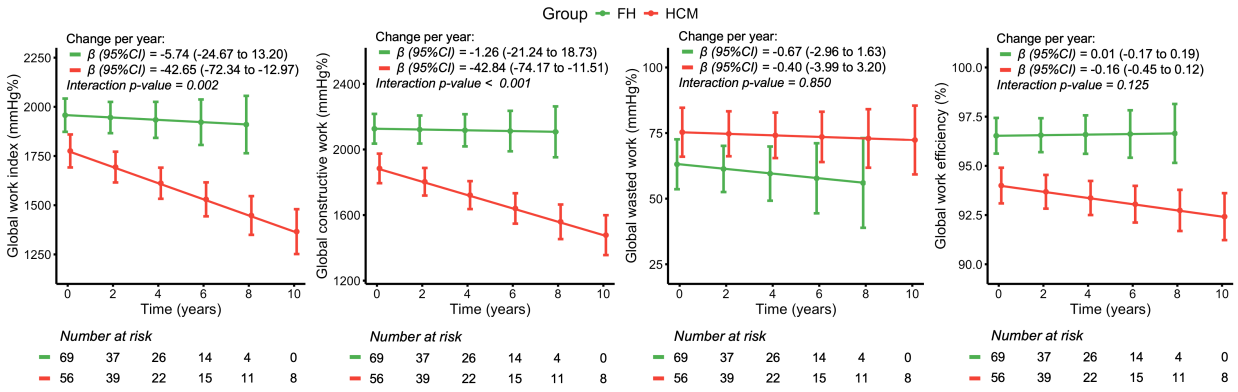

Legend: MW indices are plotted with the 95% CI at the respective time intervals. Patients with HCM (red) are compared to patients with a positive family history or a pathogenic mutation without the HCM phenotype (green). The yearly change in MW indices are expressed as β (95% CI). Abbreviations: GCW, global constructive work – GWE, global work efficiency – GWI, global work index – GWW, global wasted work.

Supplemental Figure 2. Mean change of global longitudinal strain and blood pressure over time.

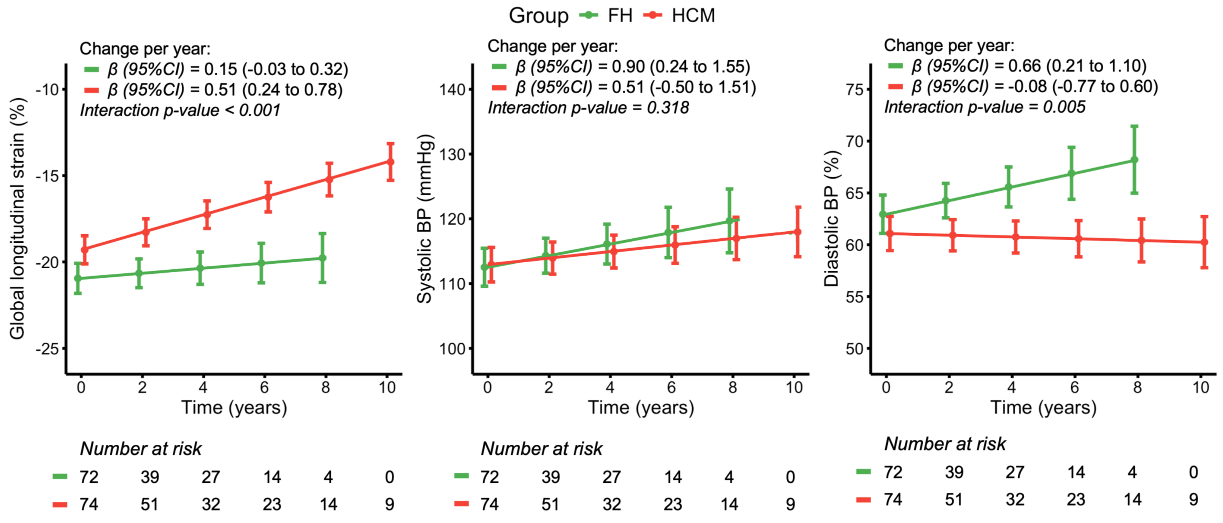

Legend: Variables are plotted with the 95% CI at the respective time intervals. Patients with HCM (red) are compared to patients with a positive family history or a pathogenic mutation without the HCM phenotype (green).
